# Risk factors associated with severe outcomes in adult hospitalized patients according to influenza type and subtype

**DOI:** 10.1371/journal.pone.0210353

**Published:** 2019-01-11

**Authors:** Ana Martínez, Núria Soldevila, Arantxa Romero-Tamarit, Núria Torner, Pere Godoy, Cristina Rius, Mireia Jané, Àngela Domínguez, and the Surveillance of Hospitalized Cases of Severe Influenza in Catalonia Working Group

**Affiliations:** 1 Agència de Salut Pública de Catalunya, Generalitat de Catalunya, Barcelona, Spain; 2 CIBER Epidemiología y Salud Pública (CIBERESP), Madrid, Spain; 3 Departament de Medicina, Universitat de Barcelona, Barcelona, Spain; 4 Agència de Salut Pública de Barcelona, Barcelona, Spain; The Ohio State University, UNITED STATES

## Abstract

Seasonal influenza is a cause of hospitalization, especially in people with underlying disease or extreme age, and its severity may differ depending on the types and subtypes of circulating viruses. We investigated the factors associated with ICU admission or death in hospitalized patients with severe laboratory-confirmed influenza according to the viral type and subtype. An observational epidemiological study was carried out in patients aged ≥18 years from 12 Catalan hospitals between 2010 and 2016. For each reported case we collected demographic, virological and clinical characteristics. A mixed-effects logistic regression model was used to estimate crude and adjusted ORs. 1726 hospitalized patients were included: 595 (34.5%) were admitted to the ICU and 224 (13.0%) died. Lower ICU admission was associated with age ≥75 years in all influenza types and subtypes and with age 65–74 years for type A. In contrast, the 65–74 and ≥75 years age groups were associated with an increased risk of death in all types and subtypes, especially for type B (aOR 27.42, 95% CI: 4.95–151.93 and 15.96; 95% CI: 3.01–84.68). The comorbidity most closely associated with severe outcomes was immune deficiency, which was associated with death for type B (aOR 9.02, 95% CI: 3.05–26.69) and subtype A(H1N1)pdm09 (aOR 3.16, 95% CI: 1.77–5.66). Older age was a differential factor for ICU admission and death: it was associated with lower ICU admission but a risk factor for death. The comorbidity with the closest association with death was immune deficiency, mainly in influenza type B patients.

## Introduction

Influenza A and B, the two influenza viruses that cause epidemics in humans, are responsible for substantial annual morbidity and mortality worldwide. Each year an estimated 10–20% of persons have influenza, although there is significant year-to-year variability in rates of illness [1]. Annual epidemics result in about 3 million to 5 million cases of severe illness and about 290,000 to 650,000 deaths worldwide [2]. Most infections are self-limited, requiring no healthcare visits, but a proportion of cases present severe complications, mainly in people with underlying health conditions and in young children and the elderly.

On the basis of antigenic differences, influenza A viruses are categorized into subtypes according to haemagglutinin and neuraminidase, the surface antigens, and influenza B viruses are separated into the Victoria and Yamagata lineages. Influenza A(H1N1)pdm09, influenza A(H3N2) and influenza B viruses co-circulate each year [3,4].

Annual excess mortality appears to have increased over recent decades, perhaps because the number of elderly and immunocompromised individuals has also increased [3], and is typically higher in A(H3N2) infections than in A(H1N1)pdm09 infections [5]. Infection due to influenza B viruses is often perceived to be milder than influenza A virus infection [6]. However, similar clinical features between outpatients infected with seasonal influenza A or B viruses and no clinical differences between influenza A(H1N1)pdm09 and A(H3N2) in the post-pandemic period have been reported [7–9]. In hospitalized patients, some independent factors have been associated with mortality and intensive care unit (ICU) admission [10,11], but studies of the factors associated with severity according to the influenza type or subtype are scarce [12]. Given the year-to-year variability in influenza severity, research on factors associated with poor outcomes for the different types and subtypes is needed to prepare ICU capacity and health resources [13,14].

In October 2010, the Public Health Agency of Catalonia initiated surveillance of severe hospitalized cases of influenza to complement the information provided by the influenza sentinel system based on primary healthcare physicians [15]. The effectiveness of antiviral treatment and vaccination in avoiding death in patients included in the surveillance system, with all types and subtypes considered jointly, has been reported previously. Receiving antiviral treatment in the first 5 days after symptom onset was effective in avoiding ICU admission and death in hospitalized patients with laboratory-confirmed influenza, and seasonal influenza vaccination was effective in preventing a composite outcome of (ICU admission or death) [16,17]. However, the effect of these control measures against poor outcomes have not been investigated in specific influenza types and subtypes.

The aim of this study was to investigate factors associated with ICU admission or death in hospitalized patients with severe laboratory-confirmed influenza according to the viral type and subtype.

## Material and methods

### Study design

An observational epidemiological case-to-case study was carried out in adult patients hospitalized due to severe acute influenza virus infection.

The surveillance system of severe hospitalized influenza in Catalonia, a region in the northeast of Spain with 7.5 million inhabitants, included 12 hospitals covering a total population of 4,644,543 (62% of the Catalan population).

A hospitalized case of severe influenza was defined as a severe case of laboratory-confirmed influenza virus infection that required hospitalization (pneumonia, septic shock, multiorgan failure or any other severe condition, including ICU admission) or who developed clinical signs during hospitalization for other reasons. The diagnosis was confirmed by PCR and/or culture of nasopharyngeal swabs.

Respiratory tract samples were processed at each hospital laboratory within 24 hours of receipt. A 300 μL aliquot was taken for total nucleic acid extraction and eluted in 25 μL of RNase-free elution buffer using the automatic QIAsymphony system (Qiagen, Hilden, Germany) according to the manufacturer's instructions. Subsequently, two specific one-step multiplex real-time PCR techniques using Stratagene Mx3000P QPCR Systems (Agilent Technologies, Santa Clara, CA, USA) were carried out to type A/B influenza viruses (sensitivity was 10 and 103 copies/μL, respectively) and influenza A virus subtypes (sensitivity was 102, 103 and 10 copies/μL for H1, H3 and H5 RNA, respectively) [18].

### Data collected

Reported cases of severe laboratory-confirmed influenza requiring hospitalization in persons aged ≥18 years in influenza seasons (2010–11 to 2015–16) were included.

For each reported case the following variables were collected: age, sex, chronic obstructive pulmonary disease (COPD), obesity (BMI >40), diabetes, chronic renal disease, immune deficiency (HIV infection or other), chronic cardiovascular disease, chronic liver disease, complications (pneumonia, acute respiratory distress syndrome [ARDS] and multiple organ failure), date of symptom onset, hospital stay, seasonal influenza vaccination status, date of antiviral treatment, ICU admission, death, and type of virus (A or B) and subtype (H1N1pdm09 or H3N2).

Cases were considered vaccinated if they had received a dose of influenza vaccine ≥14 days before symptom onset. The information on vaccination status was collected from medical records.

The information for each study variable was collected by public health officers from the surveillance units of Catalonia through an epidemiological survey. The primary source of information was the medical record.

### Statistical analysis

The demographic and clinical characteristics (independent variables) of ICU admission and non-ICU admission, and death and survival (dependent variables), were compared using the Chi square test. A p value <0.05 was considered statistically significant.

Possible interactions between independent variables were analysed by logistic regression. Independent variables were checked for collinearity using the variance inflation factor [19].

Because the participating hospitals may not have been homogeneous and there were differences in the number of ICU admissions and deaths between hospitals, a mixed-effects logistic regression model with the variable *hospital* as a random intercept was constructed to estimate the crude and adjusted odds ratios (OR) and the corresponding 95% confidence intervals (CI). Adjusted OR (aOR) were calculated using the mixed-effects logistic regression model with backward selection of variables, with a cut-off point of p<0.2, and the variable *season*, known to be a potential confounder was also included in the multivariable analysis.

The analysis was performed using the SPSS v.24 statistical package and R v3.5.0 statistical software (http://cran.r-project.org).

### Ethical considerations

All data used in the analysis were collected in the routine public health surveillance activities, as part of the legislated mandate of the Health Department of Catalonia, the competent authority for the surveillance of communicable diseases, which is officially authorized to receive, treat and temporarily store personal data on cases of infectious disease [20]. Therefore, all study activities formed part of public health surveillance and were thus exempt from institutional board review and did not require informed consent. All data were fully anonymized.

## Results

A total of 1726 hospitalized patients aged ≥18 years were included during the study period. Influenza virus type A was detected in 1483 (85.9%) patients and type B in 243 (14.1%). Of cases with type A influenza virus, 696 (46.9%) had the (H1N1)pdm09 subtype, 388 (26.2%) the A(H3N2) subtype and 399 (26.9%) were not subtyped.

Demographic and clinical characteristics for all patients, patients admitted to the ICU and deaths are shown in Tables 1–3.

**Table 1:** Characteristics of all patients hospitalized according to influenza type and subtype.

	All patients n = 1726	A* n = 1483 (85.9%)	B n = 243 (14.1%)	p value	A(H1N1)pdm09 n = 696 (40.3%)	A(H3N2) n = 388 (22.5%)	p value
**Age (years)**							
Mean ± SD	64.4±17.2	64.2±17.2	66.0±17.1	0.13	58.8±16.4	71.9±16.1	<0.01
18–64	807 (46.8%)	707 (47.7%)	100 (41.2%)	0.12	421 (60.5%)	107 (27.6%)	<0.01
65–74	328 (19.0%)	273 (18.4%)	55 (22.6%)		132 (19.0%)	65 (16.8%)	
≥75	591 (34.2%)	503 (33.9%)	88 (36.2%)		143 (20.5%)	216 (55.7%)	
**Female**	743 (43.0%)	652 (44.0%)	91 (37.4%)	0.06	285 (59.1%)	186 (47.9%)	0.03
**Male**	983 (57.0%)	831 (56.0%)	152 (62.6%)		411 (40.9%)	202 (52.1%)	
**≥1 comorbidity**	1317 (76.3%)	1134 (76.5%)	183 (75.3%)	0.69	518 (74.4%)	311 (80.2%)	0.03
**No comorbidities**	409 (23.7%)	349 (23.5%)	60 (24.7%)		178 (25.6%)	77 (19.8%)	
**COPD**	442 (25.6%)	379 (25.6%)	63 (25.9%)	0.90	169 (24.3%)	112 (28.9%)	0.10
**Obesity**	182 (10.5%)	167 (11.3%)	15 (6.2%)	0.02	86 (12.4%)	33 (8.5%)	0.05
**Diabetes**	431 (25.0%)	369 (24.9%)	62 (25.5%)	0.83	153 (22.0%)	116 (29.9%)	<0.01
**Chronic renal disease**	236 (13.7%)	196 (13.2%)	40 (16.5%)	0.17	81 (11.6%)	60 (15.5%)	0.07
**Immune deficiency**	335 (19.4%)	287 (19.4%)	48 (19.8%)	0.88	144 (20.7%)	73 (18.8%)	0.46
**Chronic cardiovascular disease**	508 (29.4%)	437 (29.5%)	71 (29.2%)	0.94	179 (25.7%)	148 (38.1%)	<0.01
**Chronic liver disease**	113 (6.5%)	93 (6.3%)	20 (8.2%)	0.25	41 (5.9%)	25 (6.4%)	0.71
**Onset of symptoms to hospitalization**				0.33			<0.01
≤2 days	703 (41.0%)	611 (41.5%)	92 (38.2%)		254 (36.9%)	189 (48.7%)	
>2 days	1010 (59.0%)	861 (58.5%)	149 (61.8%)		435 (63.1%)	199 (51.3%)	
**Antiviral treatment (since symptom onset)**							
Mean ± SD	5.1±4.6	5.0±4.6	6.0±5.0	<0.01	5.2±4.8	4.8±4.6	0.22
≤48h symptom onset	437 (25.3%)	391 (27.4%)	46 (19.9%)	<0.01	169 (24.9%)	107 (28.9%)	0.02
>48h symptom onset	1073 (62.2%)	931 (65.2%)	142 (61.5%)		467 (68.9%)	226 (61.1%)	
No	150 (8.7%)	107 (7.5%)	43 (18.6%)		42 (6.2%)	37 (10.0%)	
**Seasonal influenza vaccine**	449 (26.0%)	374 (25.5%)	75 (31.0%)	0.07	125 (18.3%)	145 (37.5%)	<0.01
**Pneumonia**	1306 (75.9%)	1113 (75.4%)	193 (79.4%)	0.17	548 (79.1%)	292 (75.3%)	0.15
**ARDS**	659 (39.0%)	580 (39.9%)	79 (33.2%)	0.05	255 (37.6%)	141 (36.7%)	0.77
**Multiorgan failure**	176 (10.5%)	151 (10.5%)	25 (10.5%)	0.99	76 (11.3%)	34 (8.9%)	0.21
**Hospital stay (days)**							
Mean ± SD	14.8±16.0	14.7±15.8	15.1±17.5	0.75	15.5±15.9	15.0±17.6	0.67
0–14 days	1194 (69.3%)	1029 (69.5%)	165 (67.9%)	0.62	469 (67.5%)	276 (71.3%)	0.19
>14 days	530 (30.7%)	452 (30.5%)	78 (32.1%)		226 (32.5%)	111 (28.7%)	
**ICU admission**	595 (34.5%)	520 (35.1%)	75 (30.9%)	0.20	276 (39.6%)	101 (26.0%)	<0.01
**Death**	224 (13.0%)	193 (13.0%)	31 (12.8%)	0.91	95 (13.6%)	56 (14.4%)	0.72
**Season**				<0.01			
2010–11	169 (9.8%)	165 (11.1%)	4 (1.6%)		153 (22.0%)	1 (0.3%)	
2011–12	122 (7.1%)	116 (7.8%)	6 (2.5%)		0 (0%)	96 (24.7%)	
2012–13	118 (6.8%)	52 (3.5%)	66 (27.2%)		35 (5.0%)	7 (1.8%)	
2013–14	345 (20.0%)	344 (23.2%)	1 (0.4%)		150 (21.6%)	134 (34.5%)	
2014–15	425 (24.6%)	364 (24.5%)	61 (25.1%)		90 (12.9%)	142 (36.6%)	
2015–16	547 (31.7%)	442 (29.8%)	105 (43.2%)		268 (38.5%)	8 (2.1%)	

*Includes types A(H1N1)pdm09, A(H3N2) and 399 not subtyped influenza A virus

COPD: chronic obstructive pulmonary disease, ARDS: acute respiratory distress syndrome

**Table 2:** Factors associated with ICU admission in hospitalized patients according to influenza type and subtype.

	ICU admitted patients/All patients (n = 595/1726)	ICU ADMITTED PATIENTS					
		A* n = 520 (35.1%)	B n = 75 (30.9%)	p value	A(H1N1)pdm09 n = 276 (39.6%)	A(H3N2) n = 101 (26.0%)	p value
**Age (years)**							
Mean ± SD	59.0±15.0	58.7±14.8	61.2±16.2	0.18	56.5±14.4	64.0±15.6	<0.01
18–64	369/807 (45.7%)	330 (63.5%)	39 (52.0%)	0.15	193 (69.9%)	44 (43.6%)	<0.01
65–74	118/328 (19.0%)	100 (19.2%)	18 (24.0%)		49 (17.8%)	26 (25.7%)	
≥75	108/591 (18.3%)	90 (17.3%)	18 (24.0%)		34 (12.3%)	31 (30.7%)	
**Female**	223/743 (30.0%)	196 (37.7%)	27 (36.0%)	0.78	108 (39.1%)	40 (39.6%)	0.93
**Male**	372/983 (37.8%)	324 (62.3%)	48 (64.0%)		168 (60.9%)	61 (60.4%)	
**≥1 comorbidity**	457/1317 (34.7%)	401 (77.1%)	56 (74.7%)	0.64	205 (74.3%)	83 (82.2%)	0.11
**No comorbidities**	138/409 (33.7%)	119 (22.9%)	19 (25.3%)		71 (25.7%)	18 (17.8%)	
**COPD**	161/422 (36.4%)	136 (26.2%)	25 (33.3%)	0.19	72 (26.1%)	28 (27.7%)	0.75
**Obesity**	80/182 (44.0%)	74 (14.2%)	6 (8.0%)	0.14	39 (14.1%)	15 (14.9%)	0.86
**Diabetes**	145/431 (33.6%)	126 (24.2%)	19 (25.3%)	0.84	53 (19.2%)	34 (33.7%)	<0.01
**Chronic renal disease**	78/236 (33.1%)	68 (13.1%)	10 (13.3%)	0.95	30 (10.9%)	13 (12.9%)	0.59
**Immune deficiency**	128/335 (38.2%)	109 (21.0%)	19 (25.3%)	0.39	70 (25.4%)	16 (15.8%)	0.05
**Chronic cardiovascular disease**	156/508 (30.7%)	143 (27.5%)	13 (17.3%)	0.06	66 (23.9%)	38 (37.6%)	0.01
**Chronic liver disease**	52/113 (46.0%)	44 (8.5%)	8 (10.7%)	0.53	15 (5.4%)	12 (11.9%)	0.03
**Onset of symptoms to hospitalization**				0.18			0.12
≤2 days	224/703 (31.9%)	201 (39.2%)	23 (31.1%)		100 (36.8%)	46 (45.5%)	
>2 days	363/1010 (35.9%)	312 (60.8%)	51 (68.9%)		172 (63.2%)	55 (54.5%)	
**Antiviral treatment (since symptom onset)**							
Mean ± SD	5.4±4.7	5.3±4.7	6.4±4.1	0.07	5.1±4.2	5.5±5.1	0.46
≤48h symptom onset	136/437 (31.1%)	125 (24.9%)	11 (15.3%)	0.01	62 (23.2%)	26 (26.3%)	0.06
>48h symptom onset	401/1073 (37.4%)	350 (69.6%)	51 (70.8%)		192 (71.9%)	62 (62.6%)	
No	38/150 (25.3%)	28 (5.6%)	10 (13.9%)		13 (4.9%)	11 (11.1%)	
**Seasonal influenza vaccine**	107/449 (23.8%)	90 (17.4%)	17 (23.0%)	0.25	35 (12.9%)	32 (31.7%)	<0.01
**Pneumonia**	451/1306 (34.5%)	395 (76.6%)	56 (74.7%)	0.72	221 (80.7%)	73 (72.3%)	0.08
**ARDS**	342/659 (51.9%)	301 (59.0%)	41 (56.9%)	0.74	167 (61.9%)	52 (52.0%)	0.09
**Multiorgan failure**	131/176 (74.4%)	113 (22.5%)	18 (25.0%)	0.63	56 (21.1%)	25 (25.3%)	0.40
**Hospital stay (days)**							
Mean ± SD	23.1±20.3	23.1±20.3	23.4±21.0	0.89	26.8±26.3	23.7±19.7	0.28
0–14 days	260/1194 (21.8%)	226 (43.6%)	34 (45.3%)	0.78	119 (43.3%)	41 (41.0%)	0.69
>14 days	333/530 (62.8%)	292 (56.4%)	41 (54.7%)		156 (56.7%)	59 (59.0%)	
**Season**				<0.01			<0.01
2010–11	66/169 (39.1%)	66 (12.7%)	0 (0%)		57 (20.7%)	0 (0%)	
2011–12	44/122 (36.1%)	41 (7.9%)	3 (4.0%)		0 (0%)	31 (30.7%)	
2012–13	44/118 (37.3%)	20 (3.8%)	24 (32.0%)		15 (5.4%)	4 (4.0%)	
2013–14	130/345 (37.7%)	130 (25.0%)	0 (0%)		73 (26.4%)	26 (25.7%)	
2014–15	132/425 (31.1%)	111 (21.3%)	21 (28.0%)		41 (14.9%)	37 (36.6%)	
2015–16	179/547 (32.7%)	152 (29.2%)	27 (36.0%)		90 (32.6%)	3 (3.0%)	

*Includes types A(H1N1)pdm09, A(H3N2) and 399 not subtyped influenza A virus

COPD: chronic obstructive pulmonary disease, ARDS: acute respiratory distress syndrome

**Table 3:** Factors associated with death in hospitalized patients according to influenza type and subtype.

	Died patients/All patients (n = 595/1726)	DIED PATIENTS					
		A* n = 193 (13.0%)	B n = 31 (12.8%)	p value	A(H1N1)pdm09 n = 95 (13.6%)	A(H3N2) n = 56 (14.4%)	p value
**Age (years)**							
Mean ± SD	69.1±15.9	68.2±16.2	74.3±12.4	0.04	62.8±16.0	78.2±11.5	<0.01
18–64	74/807 (9.2%)	72 (37.3%)	2 (6.5%)	<0.01	46 (48.4%)	7 (12.5%)	<0.01
65–74	58/328 (17.7%)	44 (22.8%)	14 (45.2%)		27 (28.4%)	10 (17.9%)	
≥75	92/591 (15.6%)	77 (39.9%)	15 (48.4%)		22 (23.2%)	39 (69.6%)	
**Female**	87/743 (11.7%)	76 (39.4%)	11 (35.5%)	0.68	34 (35.8%)	28 (50.0%)	0.09
**Male**	137/983 (13.9%)	117 (60.6%)	20 (64.5%)		61 (64.2%)	28 (50.0%)	
**≥1 comorbidity**	191/1317 (14.5%)	163 (84.5%)	28 (90.3%)	0.39	81 (85.3%)	48 (85.7%)	0.94
**No comorbidities**	33/409 (8.1%)	30 (15.5%)	3 (9.7%)		14 (14.7%)	8 (14.3%)	
**COPD**	67/422 (15.2%)	56 (29.0%)	11 (35.5%)	0.46	26 (27.4%)	20 (35.7%)	0.28
**Obesity**	24/182 (13.2%)	21 (10.9%)	3 (9.7%)	0.84	10 (10.5%)	6 (10.7%)	0.97
**Diabetes**	60/431 (13.9%)	51 (26.4%)	9 (29.0%)	0.76	24 (25.3%)	15 (26.8%)	0.84
**Chronic renal disease**	49/236 (20.8%)	38 (19.7%)	11 (35.5%)	0.04	15 (15.8%)	13 (23.2%)	0.26
**Immune deficiency**	77/335 (23.0%)	64 (33.2%)	13 (41.9%)	0.34	38 (40.0%)	14 (25.0%)	0.06
**Chronic cardiovascular disease**	84/508 (16.5%)	72 (37.3%)	12 (38.7%)	0.88	36 (37.9%)	26 (46.4%)	0.30
**Chronic liver disease**	25/113 (22.1%)	23 (11.9%)	2 (6.5%)	0.37	12 (12.6%)	5 (8.9%)	0.49
**Onset of symptoms to hospitalization**				0.09			0.01
≤2 days	93/703 (13.2%)	76 (40.0%)	17 (56.7%)		29 (31.5%)	30 (53.6%)	
>2 days	127/1010 (12.6%)	114 (60.0%)	13 (43.3%)		63 (68.5%)	26 (46.4%)	
**Antiviral treatment (since symptom onset)**							
Mean ± SD	6.2±6.1	6.3±6.2	5.7±5.1	0.69	5.5±4.8	6.9±7.0	0.24
≤48h symptom onset	37/437 (8.5%)	31 (16.7%)	6 (20.7%)	0.54	11 (12.1%)	10 (18.5%)	0.01
>48h symptom onset	145/1073 (13.5%)	128 (68.8%)	17 (58.6%)		71 (78.0%)	30 (55.6%)	
No	33/150 (22.0%)	27 (14.5%)	6 (20.7%)		9 (9.9%)	14 (25.9%)	
**Seasonal influenza vaccine**	63/449 (14.0%)	53 (32.3%)	10 (27.6%)	0.59	18 (19.1%)	22 (39.3%)	0.01
**Pneumonia**	182/1306 (13.9%)	162 (84.4%)	20 (64.5%)	0.01	80 (85.1%)	46 (82.1%)	0.63
**ARDS**	134/659 (20.3%)	117 (61.6%)	17 (54.8%)	0.48	57 (61.3%)	29 (52.7%)	0.31
**Multiorgan failure**	95/176 (54.0%)	82 (43.9%)	13 (41.9%)	0.84	44 (47.8%)	18 (33.3%)	0.09
**Hospital stay (days)**							
Mean ± SD	14.6±14.9	14.4±14.9	15.6±15.2	0.67	15.6±16.9	13.6±13.0	0.42
0–14 days	144/1194 (12.1%)	124 (64.2%)	20 (64.5%)	0.98	63 (66.3%)	35 (62.5%)	0.63
>14 days	80/530 (15.1%)	69 (35.8%)	11 (35.5%)		32 (33.7%)	21 (37.5%)	
**Season**				<0.01			<0.01
2010–11	29/169 (17.2%)	27 (14.0%)	2 (6.5%)		26 (27.4%)	0 (0.0%)	
2011–12	15/122 (12.3%)	15 (7.8%)	0 (0.0%)		0 (0.0%)	14 (25.0%)	
2012–13	16/118 (13.6%)	5 (2.6%)	11 (35.5%)		4 (4.2%)	0 (0.0%)	
2013–14	43/345 (12.5%)	43 (22.3%)	0 (0.0%)		17 (17.9%)	18 (32.1%)	
2014–15	61/425 (14.4%)	51 (26.4%)	10 (32.3%)		13 (13.7%)	22 (39.3%)	
2015–16	60/547 (11.0%)	52 (26.9%)	8 (25.8%)		35 (36.8%)	2 (3.6%)	

*Includes types A(H1N1)pdm09, A(H3N2) and 399 not subtyped influenza A virus

COPD: chronic obstructive pulmonary disease, ARDS: acute respiratory distress syndrome

There was no collinearity or interaction between variables.

### Influenza type A

**Fig 1:**
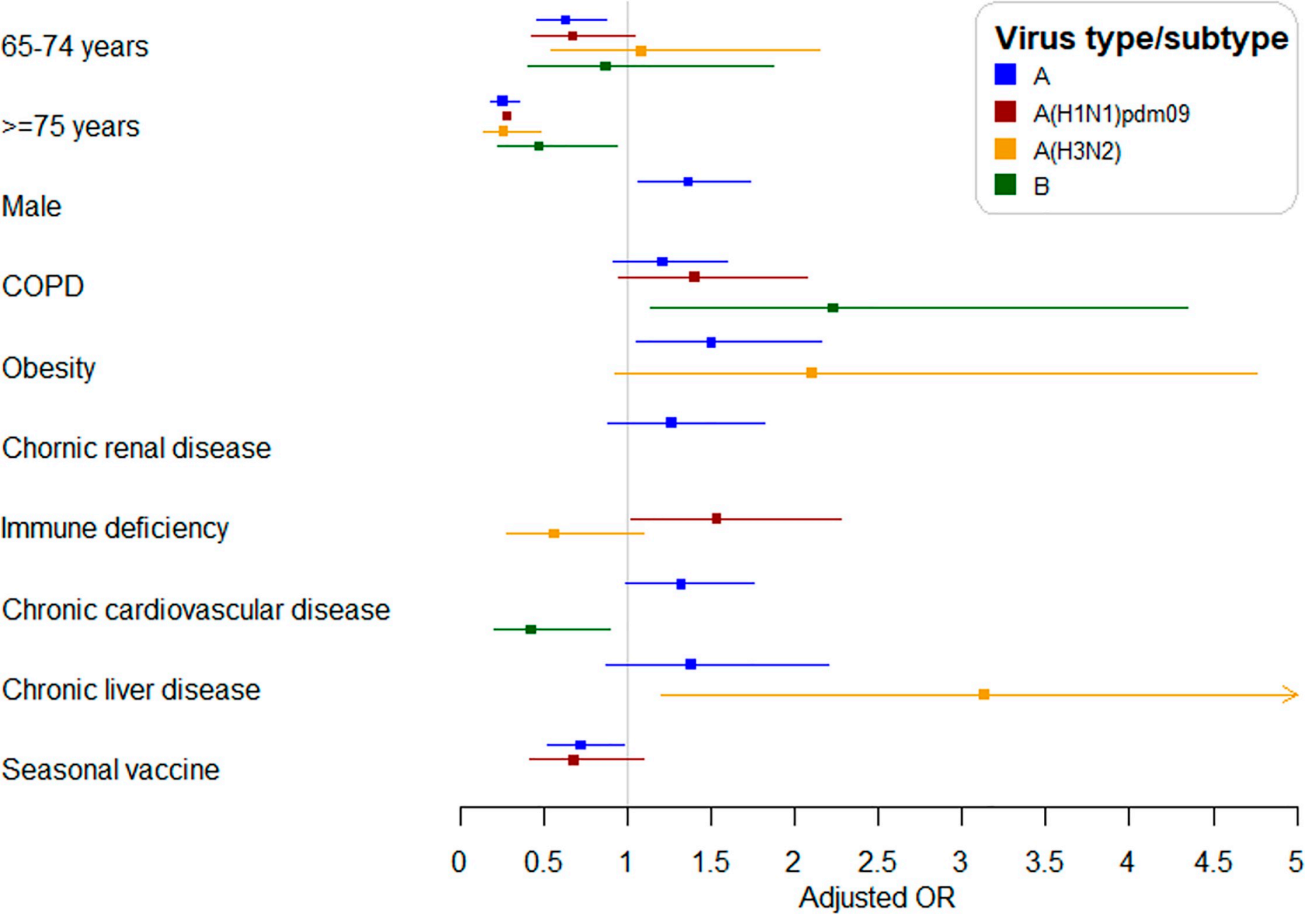
Factors associated with ICU admission in hospitalized patients according to influenza type and subtype.

A total of 520 (35.1%) patients required ICU admission and 193 (13.0%) died. In the multivariable analysis, age 65–74 and ≥75 years was associated with lower ICU admission (aOR 0.63, 95% CI: 0.46–0.87 and 0.25, 95% CI: 0.18–0.35, respectively). Male sex and obesity were associated with ICU admission and seasonal influenza vaccination was associated with lower ICU admission (aOR 0.72, 95% CI: 0.52–0.98) (Fig 1 and S1 Table).

**Fig 2:**
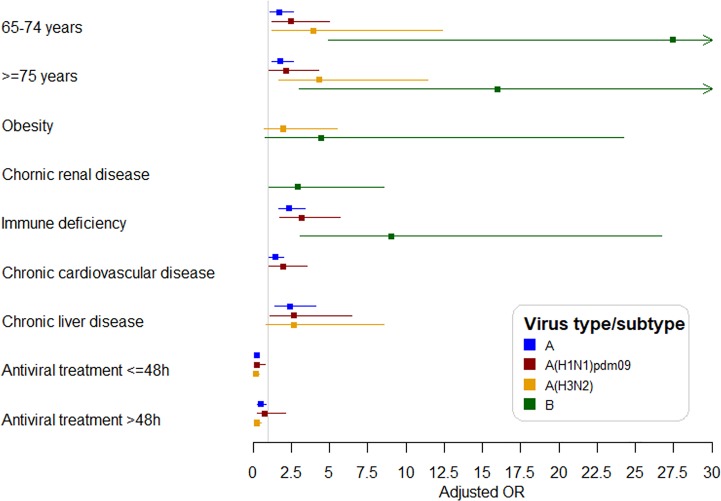
Factors associated with death in hospitalized patients according to influenza type and subtype.

The 65–74 and ≥75 years age groups were associated with an increased risk of death (aOR 1.69, 95% CI: 1.09–2.62 and 1.78, 95% CI: 1.20–2.65, respectively). Immune deficiency, chronic cardiovascular disease and chronic liver disease were also associated with an increased risk of death. Antiviral treatment was associated with a lower risk of death (Fig 2 and S2 Table).

### Influenza A(H1N1)pdm09 subtype

A total of 276 (39.6%) patients required ICU admission and 95 (13.6%) died. In the multivariable analysis, age ≥75 years was associated with lower ICU admission (aOR 0.36, 95% CI: 0.22–0.59). The 65–74 years age group was not associated with ICU admission, but the statistical power was low (43%). Immune deficiency was also associated with ICU admission (Fig 1 and S1 Table).

The 65–74 and ≥75 years age groups were associated with an increased risk of death (aOR 2.46, 95% CI: 1.22–4.97 and 2.13, 95% CI: 1.05–4.30, respectively). Immune deficiency, chronic cardiovascular disease and chronic liver disease were also associated with an increased risk of death. Antiviral treatment administered ≤48 hours before symptom onset was associated with a lower risk of death (Fig 2 and S2 Table).

### Influenza A(H3N2) subtype

A total of 101 (26.0%) patients required ICU admission and 56 (14.4%) died. In the multivariable analysis, age ≥75 years was associated with lower ICU admission (aOR 0.26, 95% CI: 0.14–0.48). The 65–74 years age group was not associated with ICU admission, but the statistical power was low (4%). Chronic liver disease was also associated with ICU admission (Fig 1 and S1 Table).

The 65–74 and ≥75 years age groups were associated with an increased risk of death (aOR 3.95, 95% CI: 1.26–12.37 and 4.35, 95% CI: 1.66–11.43, respectively). Antiviral treatment was associated with a lower risk of death (Fig 2 and S2 Table).

### Influenza type B

A total of 75 (30.9%) patients required ICU admission and 31 (12.8%) died. In the multivariable analysis, age ≥75 years was associated with lower ICU admission (aOR 0.47, 95% CI: 0.23–0.94). The 65–74 years age group was not associated with ICU admission, but the statistical power was low (12%). COPD was also associated with ICU admission and chronic cardiovascular disease was associated with a lower ICU admission (aOR 0.42, 95% CI: 0.20–0.89) (Fig 1 and S1 Table).

The 65–74 and ≥75 years age groups were associated with an increased risk of death (aOR 27.42, 95% CI: 4.95–151.93 and 15.96, 95% CI: 3.01–84.68, respectively). Chronic renal disease and immune deficiency were also associated with an increased risk of death (Fig 2 and S2 Table).

## Discussion

In this series of hospitalized cases of severe influenza recorded from 2010 to 2016 in Catalonia, subtype A(H1N1)pdm09 was the most frequent (40.3%) followed (22.5%) by influenza A(H3N2), while influenza type B was less frequent (14.1%). This distribution is similar to that reported in an Italian study from 2010–11 to 2014–15 (79.1%, 9.8% and 6.6%, respectively) and an international cohort from 2009 to 2015 (45.9%, 38.1% and 16.1%, respectively) [11,21], with the non-subtyped influenza A strains in our study (23.1%) representing an intermediate proportion of those recorded in these studies.

Patient with A(H1N1)pdm09 required ICU admission more frequently (39.6%), followed by influenza B patients (30.9%) and A(H3N2) patients (26%). The same order of frequency was registered in the international study carried out during six influenza seasons by Dwyer et al. [21] (5.9%, 5.4% and 3.6%), but in studies carried out in England during five seasons [22] and in France during three seasons [9] influenza B required a slightly higher frequency of ICU admission than subtype A(H3N2) (10.7% vs 10.5% and 14% vs 11%, respectively). The higher proportions of ICU admissions in our study may be due to the fact that only severe hospitalized cases were included rather than all the hospitalized cases included in the above mentioned studies.

Subtype A(H3N2) had the greatest mortality rate (14.4%), but the differences with respect to subtype A(H1N1)pdm09 and type B were not statistically significant, similar to other studies [9,19,23].

Patients aged ≥75 years were less frequently admitted to the ICU than those aged 18–64 years in all influenza types/subtypes and the 65–74 years age group was also associated with a lower frequency of ICU admission for all type A patients but the statistical power was low for influenza A subtypes and influenza type B. These results contrast with those related to death, which was higher in patients aged 65–74 and ≥75 years for all influenza types and subtypes. These differences suggest that age is probably taken into account when deciding whether a severe case should be admitted to the ICU. Possible explanations are that the elderly may be admitted to hospital more frequently with clinically less-severe influenza or, due to personal choice might be less frequently transferred to the ICU [13], and that younger adults are less likely to have serious comorbidities and are more likely to be admitted to the ICU if complications appear [19].

The association of the 65–74 and ≥75 years age groups with death was closer for type B and subtype A(H3N2), in agreement with the results of other studies that found high excess mortality in patients aged >70 years in years with a high rate of A(H3N2) circulation and which recommended paying more attention to this subtype in the elderly [5], and with studies that found that older age was associated with influenza type B [21,23]. Studies focused only on A(H1N1)pdm09 in Spain and in a cohort study including several countries also found a high risk of death in elderly patients [24,25]. An increased risk of death in older age groups has been also reported in a study that did not differentiate by type/subtypes [26].

Men were more frequently admitted to the ICU than women (37.8% vs. 30.0%) but this association was only significant for type A, with the other types/subtypes having insufficient statistical power. These results are in agreement with those of the crude analysis in a 2010–2012 Canadian study with 62.7% of influenza A cases although the association was lost in the adjusted analysis [13]. In our study, as in others [9,25], sex was not associated with death for any influenza type or subtype. In contrast, in a study carried out in Taiwan over three seasons, male sex was associated with an increased risk of death in patients with A(H1N1) but not in those with A(H3N2) or B virus infection [27]. As Klein et al. [28] reported, male-female differences in studies of influenza may be confounded by age and health seeking. We can reasonably rule out confounding by age because we introduced this variable in the adjusted analysis, but unfortunately we have no information about the health-seeking behaviours of patients included.

In our study, 161 patients requiring ICU care had COPD, but this was statistically significant only for type B. We have not found specific type/subtype analyses in other studies. Without distinguishing by type/subtype, Taylor et al. [13] found that COPD was more frequent in ICU patients than in non-ICU patients (42.7% vs 30.3%) and Garg et al. [29] found an association between COPD and ICU admission or death. In a study of severe outcomes in hospitalized patients with pneumonia and influenza between 2000 and 2012, COPD was associated with an increased risk of ICU admission or death in the univariate but not in the multivariate analysis [30]. Other studies found no association with death [23,24,31,32].

Obesity was associated with ICU admission in influenza A patients. Other studies in the post-pandemic period found not association between obesity and ICU admission [13,33]. A possible explanation is that, as in the case of age, obesity is taken into account when deciding whether a severe case should be admitted to the ICU. No association was found with death for any type or subtype, in agreement with the results of studies of influenza B and influenza A [23]. Specific studies of severe influenza A(H1N1)pdm09 or A(H3N2) also found no association [24,33]. A meta-analysis suggested that data on obesity and the risk of death should be interpreted with caution, because early antiviral treatment might be a key confounding factor [34].

Patients with chronic renal disease may have higher risk of poor outcomes given their altered response. We found that chronic renal disease was associated with death in influenza type B cases but not in cases of A(H1N1)pdm09 or A(H3N2). In a Spanish study of hospitalized A(H1N1)pdm09 patients, chronic renal disease was also associated with death [24], but another Spanish study [23] found no association in influenza B or influenza A(H1N1)pdm09 patients. In the study by Van Kerkhove et al. [30] in hospitalized patients with influenza and pneumonia between 2000 and 2012, chronic renal disease was associated with severity.

We found that immune deficiency was associated with ICU admission and death in influenza A(H1N1)pdm09 patients. Lynfield et al. found that immune deficiency was the only comorbidity associated with death in hospitalized A(H1N1)pdm09 patients [25] and Alvarez Lerma et al. [24] found an association between A(H1N1)pdm09 patients who died and immunosuppression. In studies that did not separate by type/subtype, an association was found between immunosuppression and ICU admission or death [13,29].

Chronic cardiovascular diseases were a protective factor against ICU admission in influenza B patients and were a risk factor for death in influenza A(H1N1)pdm09 patients. The surprising finding of the protective role of cardiovascular disease against ICU admission is similar to that found by Taylor et al. [13] in the pandemic period but not in the post-pandemic period. The evident benefits of statins in cardiovascular disease might also extend to patients with influenza [35]. Statins are widely recommended in patients with high levels of LDL-cholesterol, diabetes mellitus and a risk of cardiovascular morbidity [36]. Because these diseases are highly prevalent and the use of statins alone or in combination with other drugs is frequent in Spain [37], a possible explanation for this association might be that, in influenza B patients, a history of treatment with statins was more frequent than in influenza A patients. However, we have no information about previous statins uses and are thus unable to explain these results.

Chronic liver disease was associated with ICU admission in influenza A(H3N2) patients and with death in influenza A(H1N1)pdm09 patients, coinciding with the results of another study that did not differentiate between types or subtypes [30].

Antiviral treatment administered ≤48 hours after symptom onset prevented death in all influenza A patients considered jointly and also in A(H1N1)pdm09 and A(H3N2) patients considered separately. In influenza B patients, the statistical power was insufficient to detect associations. However, as in studies in Italy and France, fewer influenza B patients received early antiviral treatment than those with influenza A subtypes, supporting the idea that treatment should be reinforced for type B patients [11,12]. Our results on the association between early antiviral treatment and survival are similar to those found in studies in influenza A(H1N1) patients [24,27] and a study that did not separate by type/subtypes but with a large majority of influenza A cases [26]. This suggests that antiviral treatment is useful in reducing mortality in all types and subtypes and that early treatment should be reinforced.

Seasonal influenza vaccination was associated with a reduced risk of ICU admission in influenza A patients considered globally, but the statistical power was low to detect any association in type B or in A subtypes. In the Canadian study by Taylor et al. [13] in the post-pandemic period in which most cases were due to influenza A, vaccination was not associated with reduced ICU admission. Like other studies [9,19,38], we found differences in the vaccination coverage according to type/subtypes: the highest coverage (37.5%) was in A(H3N2) patients, followed by influenza B and influenza A(H1N1)pdm09 patients (31.0% and 18.3%), suggesting lower effectiveness against subtype A(H3N2), which may be explained by more frequent mutations in this subtype and difficulties in obtaining the corresponding strain for the vaccine [1,39].

Rizzo et al. [11] reported that, of the 72% of influenza B patients with comorbidities that are indications for seasonal vaccination, only 22% were vaccinated, figures close to ours. We agree that interventions against B type using antiviral treatment and vaccination should be improved.

This study, like all observational studies, has strengths and limitations. First, comparative studies between factors associated with severe outcomes according to influenza type and subtype are scarce and, therefore, our results may help identify them. Secondly, unlike studies based on nonspecific outcomes [5,30], all cases included were laboratory-confirmed and, therefore, the distribution of independent variables corresponds to real severe influenza cases. Thirdly, the sample size permitted a multivariable analysis, therefore reducing the possibility of confounding factors invalidating the results.

The limitations include, firstly, the voluntary participation of hospitals, which could lead to selection bias. However, because the hospitals participating in the surveillance system cover more than 60% of the Catalan population and a mixed-effect logistic regression model with hospitals as a random intercept was used, we believe our results might be extensible to severe hospitalized patients in Catalonia. Secondly, not all influenza A case virus were subtyped. However, because the number of influenza A(H1N1)pdm09 and A(H3N2) cases was high, conclusions about the differences in risk factors related to these two subtypes seem robust. Thirdly, we were not able to investigate differences between the Victoria and Yamagata lineages because this information was not available. Fourthly, all comparisons were between severe hospitalized cases, and the results cannot be extrapolated to non-severe influenza cases.

In conclusion, although possible explanations are not always available, our results suggest that predictors of poor outcomes of influenza may vary by type/subtypes. Older age was a differential factor in patients hospitalized due to severe influenza with respect to ICU admission and death. While age ≥65 years was a risk factor for death in all influenza types and subtypes, and especially for type B, age >75 years was associated with lower ICU admission for all influenza types and subtypes. The comorbidity with the closest association with death was immune deficiency, mainly in type B patients. Further studies on the virology of hospitalized influenza patients are required.

## Supporting information

S1 Table Factors associated with ICU admission in hospitalized patients according to influenza type and subtype. (DOC)

S2 Table Factors associated with death in hospitalized patients according to influenza type and subtype. (DOC)
