# Correction: Risk factors associated with severe outcomes in adult hospitalized patients according to influenza type and subtype

**DOI:** 10.1371/journal.pone.0314310

**Published:** 2024-11-19

**Authors:** Ana Martínez, Núria Soldevila, Arantxa Romero-Tamarit, Núria Torner, Pere Godoy, Cristina Rius, Mireia Jané, Àngela Domínguez and the Surveillance of Hospitalized Cases of Severe Influenza in Catalonia Working Group

In Table 3, the value of n for the heading Died patients/ All patients is incorrect. The correct value is: n = 224/1726. Please see the correct Table 3 here.

**Table 3:** Factors associated with death in hospitalized patients according to influenza type and subtype.

	Died patients/All patients (n = 224/1726)	DIED PATIENTS					
		A* n = 193 (13.0%)	B n = 31 (12.8%)	p value	A(H1N1)pdm09 n = 95 (13.6%)	A(H3N2) n = 56 (14.4%)	p value
**Age (years)**							
Mean ± SD	69.1±15.9	68.2±16.2	74.3±12.4	0.04	62.8±16.0	78.2±11.5	<0.01
18–64	74/807 (9.2%)	72 (37.3%)	2 (6.5%)	<0.01	46 (48.4%)	7 (12.5%)	<0.01
65–74	58/328 (17.7%)	44 (22.8%)	14 (45.2%)		27 (28.4%)	10 (17.9%)	
≥75	92/591 (15.6%)	77 (39.9%)	15 (48.4%)		22 (23.2%)	39 (69.6%)	
**Female**	87/743 (11.7%)	76 (39.4%)	11 (35.5%)	0.68	34 (35.8%)	28 (50.0%)	0.09
**Male**	137/983 (13.9%)	117 (60.6%)	20 (64.5%)		61 (64.2%)	28 (50.0%)	
**≥1 comorbidity**	191/1317 (14.5%)	163 (84.5%)	28 (90.3%)	0.39	81 (85.3%)	48 (85.7%)	0.94
**No comorbidities**	33/409 (8.1%)	30 (15.5%)	3 (9.7%)		14 (14.7%)	8 (14.3%)	
**COPD**	67/422 (15.2%)	56 (29.0%)	11 (35.5%)	0.46	26 (27.4%)	20 (35.7%)	0.28
**Obesity**	24/182 (13.2%)	21 (10.9%)	3 (9.7%)	0.84	10 (10.5%)	6 (10.7%)	0.97
**Diabetes**	60/431 (13.9%)	51 (26.4%)	9 (29.0%)	0.76	24 (25.3%)	15 (26.8%)	0.84
**Chronic renal disease**	49/236 (20.8%)	38 (19.7%)	11 (35.5%)	0.04	15 (15.8%)	13 (23.2%)	0.26
**Immune deficiency**	77/335 (23.0%)	64 (33.2%)	13 (41.9%)	0.34	38 (40.0%)	14 (25.0%)	0.06
**Chronic cardiovascular disease**	84/508 (16.5%)	72 (37.3%)	12 (38.7%)	0.88	36 (37.9%)	26 (46.4%)	0.30
**Chronic liver disease**	25/113 (22.1%)	23 (11.9%)	2 (6.5%)	0.37	12 (12.6%)	5 (8.9%)	0.49
**Onset of symptoms to hospitalization**				0.09			0.01
≤2 days	93/703 (13.2%)	76 (40.0%)	17 (56.7%)		29 (31.5%)	30 (53.6%)	
>2 days	127/1010 (12.6%)	114 (60.0%)	13 (43.3%)		63 (68.5%)	26 (46.4%)	
**Antiviral treatment (since symptom onset)**							
Mean ± SD	6.2±6.1	6.3±6.2	5.7±5.1	0.69	5.5±4.8	6.9±7.0	0.24
≤48h symptom onset	37/437 (8.5%)	31 (16.7%)	6 (20.7%)	0.54	11 (12.1%)	10 (18.5%)	0.01
>48h symptom onset	145/1073 (13.5%)	128 (68.8%)	17 (58.6%)		71 (78.0%)	30 (55.6%)	
No	33/150 (22.0%)	27 (14.5%)	6 (20.7%)		9 (9.9%)	14 (25.9%)	
**Seasonal influenza vaccine**	63/449 (14.0%)	53 (32.3%)	10 (27.6%)	0.59	18 (19.1%)	22 (39.3%)	0.01
**Pneumonia**	182/1306 (13.9%)	162 (84.4%)	20 (64.5%)	0.01	80 (85.1%)	46 (82.1%)	0.63
**ARDS**	134/659 (20.3%)	117 (61.6%)	17 (54.8%)	0.48	57 (61.3%)	29 (52.7%)	0.31
**Multiorgan failure**	95/176 (54.0%)	82 (43.9%)	13 (41.9%)	0.84	44 (47.8%)	18 (33.3%)	0.09
**Hospital stay (days)**							
Mean ± SD	14.6±14.9	14.4±14.9	15.6±15.2	0.67	15.6±16.9	13.6±13.0	0.42
0–14 days	144/1194 (12.1%)	124 (64.2%)	20 (64.5%)	0.98	63 (66.3%)	35 (62.5%)	0.63
>14 days	80/530 (15.1%)	69 (35.8%)	11 (35.5%)		32 (33.7%)	21 (37.5%)	
**Season**				<0.01			<0.01
2010–11	29/169 (17.2%)	27 (14.0%)	2 (6.5%)		26 (27.4%)	0 (0.0%)	
2011–12	15/122 (12.3%)	15 (7.8%)	0 (0.0%)		0 (0.0%)	14 (25.0%)	
2012–13	16/118 (13.6%)	5 (2.6%)	11 (35.5%)		4 (4.2%)	0 (0.0%)	
2013–14	43/345 (12.5%)	43 (22.3%)	0 (0.0%)		17 (17.9%)	18 (32.1%)	
2014–15	61/425 (14.4%)	51 (26.4%)	10 (32.3%)		13 (13.7%)	22 (39.3%)	
2015–16	60/547 (11.0%)	52 (26.9%)	8 (25.8%)		35 (36.8%)	2 (3.6%)	
