# Supplementary material for: Risk factors associated with severe outcomes in adult hospitalized patients according to influenza type and subtype
Source: PLoS One. 2019 Jan 11;14(1):e0210353. doi: 10.1371/journal.pone.0210353 (PMC6329503; doi:10.1371/journal.pone.0210353)
Supplement: S1 Table — (DOC) [file pone.0210353.s001.doc]

**S1 Table**. Factors associated with ICU admission in hospitalized patients according to influenza type and subtype

|  | **A** | | **A(H1N1)pdm09** | | **A(H3N2)** | | **B** | |
| --- | --- | --- | --- | --- | --- | --- | --- | --- |
|  | **Crude OR**  **(95% CI)** | **Adjusted OR (95% CI)** | **Crude OR**  **(95% CI)** | **Adjusted OR**  **(95% CI)** | **Crude OR**  **(95% CI)** | **Adjusted OR**  **(95% CI)** | **Crude OR**  **(95% CI)** | **Adjusted OR**  **(95% CI)** |
| **Age (years)** |  |  |  |  |  |  |  |  |
| 18-64 | Ref. | Ref. | Ref. | Ref. | Ref. | Ref. | Ref. | Ref. |
| 65-74 | **0.68 (0.50-0.91)** | **0.63 (0.46-0.87)** | 0.70 (0.47-1.06)^a^ | 0.67 (0.43-1.04) | 0.96 (0.50-1.82)^b^ | 1.08 (0.54-2.15) | 0.76 (0.38-1.52)^c^ | 0.87 (0.41-1.87) |
| ≥75 | **0.27 (0.20-0.36)** | **0.25 (0.18-0.35)** | **0.37 (0.24-0.57)** | **0.36 (0.22-0.59)** | **0.23 (0.13-0.40)** | **0.26 (0.14-0.48)** | **0.40 (0.21-0.78)** | **0.47 (0.23-0.94)** |
| **Male** | **1.44 (1.15-1.81)** | **1.36 (1.07-1.73)** | 1.13 (0.83-1.55)^d^ |  | 1.54 (0.96-2.45)^e^ |  | 1.10 (0.62-1.95)^f^ |  |
| **COPD** | 1.08 (0.84-1.39) | 1.21 (0.92-1.60) | 1.17 (0.82-1.68) | 1.40 (0.95-2.07) | 0.94 (0.56-1.57) |  | 1.70 (0.93-3.11) | **2.23 (1.14-4.35)** |
| **Obesity** | **1.61 (1.15-2.26)** | **1.50 (1.05-2.16)** | 1.32 (0.83-2.10) |  | **2.67 (1.27-5.63)** | 2.10 (0.93-4.73) | 1.49 (0.50-4.46) |  |
| **Diabetes** | 0.95 (0.74-1.22) |  | 0.78 (0.53-1.13) |  | 1.23 (0.75-2.01) |  | 0.98 (0.52-1.84) |  |
| **Chronic renal disease** | 0.99 (0.72-1.38) | 1.26 (0.88-1.82) | 0.90 (0.55-1.46) |  | 0.77 (0.39-1.50) |  | 0.67 (0.30-1.49) |  |
| **Immune deficiency** | 1.11 (0.84-1.47) |  | **1.57 (1.08-2.30)** | **1.53 (1.02-2.28)** | 0.80 (0.43-1.48) | 0.56 (0.28-1.10) | 1.61 (0.83-3.14) |  |
| **Chronic cardiovascular disease** | 0.91 (0.71-1.16)^g^ | 1.32 (0.99-1.76) | 0.90 (0.63-1.29)^h^ |  | 0.97 (0.60-1.56)^i^ |  | **0.40 (0.20-0.78)** | **0.42 (0.20-0.89)** |
| **Chronic liver disease** | **1.68 (1.09-2.61)**^j^ | 1.38 (0.87-2.20) | 0.88 (0.45-1.71)^k^ |  | **3.62 (1.50-8.72)** | **3.13 (1.20-8.14)** | 1.52 (0.59-3.94)^l^ |  |
| **Onset of symptoms to hospitalization** |  |  |  |  |  |  |  |  |
| ≤2 days | Ref. |  | Ref. |  | Ref. |  | Ref. |  |
| >2 days | 1.05 (0.84-1.32) |  | 0.98 (0.71-1.36) |  | 1.17 (0.74-1.86) |  | 1.58 (0.88-2.85) |  |
| **Antiviral treatment** |  |  |  |  |  |  |  |  |
| ≤48h symptom onset | 1.59 (0.97-2.62) |  | 1.36 (0.65-2.85) |  | 0.81 (0.34-1.94) |  | 1.03 (0.39-2.76) |  |
| >48h symptom onset | **1.74 (1.10-2.76)** |  | 1.54 (0.78-3.07) |  | 0.94 (0.43-2.05) |  | 1.86 (0.84-4.12) |  |
| No | Ref. |  | Ref. |  | Ref. |  | Ref. |  |
| **Seasonal influenza vaccine** | **0.51 (0.39-0.67)** | **0.72 (0.52-0.98)** | **0.52 (0.34-0.80)** | 0.68 (0.42-1.10)^m^ | 0.67 (0.41-1.10)^n^ |  | 0.57 (0.30-1.08)^o^ |  |

Figures in bold show statistically-significant differences (p<0.05)

Statistical power: ^a^ 43%, ^b^ 4%, ^c^ 12%, ^d^ 12%, ^e^ 50%, ^f^ 5%, ^g^ 23%, ^h^ 14%, ^i^ 4%, ^j^ 68%, ^k^ 6%, ^l^ 15%, ^m^50%, ^n^29%, ^o^44%

COPD: chronic obstructive pulmonary disease
