# Supplementary material for: Risk factors associated with severe outcomes in adult hospitalized patients according to influenza type and subtype
Source: PLoS One. 2019 Jan 11;14(1):e0210353. doi: 10.1371/journal.pone.0210353 (PMC6329503; doi:10.1371/journal.pone.0210353)
Supplement: S2 Table — (DOC) [file pone.0210353.s002.doc]

**S2 Table**. Factors associated with death in hospitalized patients according to influenza type and subtype

|  | **A** | | **A(H1N1)pdm09** | | **A(H3N2)** | | **B** | |
| --- | --- | --- | --- | --- | --- | --- | --- | --- |
|  | **Crude OR**  **(95% CI)** | **Adjusted OR (95% CI)** | **Crude OR**  **(95% CI)** | **Adjusted OR**  **(95% CI)** | **Crude OR**  **(95% CI)** | **Adjusted OR**  **(95% CI)** | **Crude OR**  **(95% CI)** | **Adjusted OR**  **(95% CI)** |
| **Age (years)** |  |  |  |  |  |  |  |  |
| 18-64 | Ref. | Ref. | Ref. | Ref. | Ref. | Ref. | Ref. | Ref. |
| 65-74 | **1.71 (1.14-2.57)** | **1.69 (1.09-2.62)** | **2.07 (1.22-3.51)** | **2.46 (1.22-4.97)** | 2.60 (0.94-7.21) | **3.95 (1.26-12.37)** | **18.83 (4.04-87.69)** | **27.42 (4.95-151.93)** |
| ≥75 | **1.66 (1.17-2.36)** | **1.78 (1.20-2.65)** | 1.42 (0.82-2.48) | **2.13 (1.05-4.30)** | **3.15 (1.36-7.30)** | **4.35 (1.66-11.43)** | **10.56 (2.33-47.83)** | **15.96 (3.01-84.68)** |
| **Male** | 1.24 (0.91-1.69) |  | 1.27 (0.80-1.99) |  | 0.91 (0.52-1.60) |  | 1.20 (0.53-2.68) |  |
| **COPD** | 1.25 (0.89-1.75) |  | 1.22 (0.74-1.99) |  | 1.45 (0.80-2.63) |  | 1.68 (0.75-3.80) |  |
| **Obesity** | 0.99 (0.61-1.62) |  | 0.81 (0.40-1.65) |  | 1.36 (0.53-3.45) | 1.98 (0.72-5.48) | 1.53 (0.38-6.11) | 4.42 (0.81-24.21) |
| **Diabetes** | 1.10 (0.78-1.56) |  | 1.25 (0.75-2.08) |  | 0.84 (0.44-1.58) |  | 1.31 (0.55-3.08) |  |
| **Chronic renal disease** | **1.69 (1.14-2.51)** |  | 1.38 (0.74-2.57) |  | 1.83 (0.92-3.66) |  | **3.22 (1.37-7.59)** | **2.93 (1.01-8.53)** |
| **Immune deficiency** | **2.21 (1.56-3.12)** | **2.37 (1.66-3.39)** | **2.93 (1.82-4.72)** | **3.16 (1.77-5.66)** | 1.54 (0.79-3.00) |  | **3.32 (1.43-7.75)** | **9.02 (3.05-26.69)** |
| **Chronic cardiovascular disease** | **1.45 (1.05-2.00)** | **1.43 (1.01-2.02)** | **1.93 (1.21-3.07)** | **1.94 (1.07-3.54)** | 1.49 (0.84-2.64) |  | 1.57 (0.71-3.49) |  |
| **Chronic liver disease** | **2.10 (1.26-3.49)** | **2.40 (1.41-4.08)** | **2.51 (1.21-5.24)** | **2.68 (1.11-6.48)** | 1.53 (0.55-4.26) | 2.65 (0.82-8.57) | 0.64 (0.14-2.96) |  |
| **Onset of symptoms to hospitalization** |  |  |  |  |  |  |  |  |
| ≤2 days | Ref. |  | Ref. |  | Ref. |  | Ref. | Ref. |
| >2 days | 1.02 (0.75-1.40) |  | 1.32 (0.82-2.13) |  | 0.80 (0.45-1.40) |  | 0.45 (0.21-1.00) | 0.57 (0.22-1.45) |
| **Antiviral treatment** |  |  |  |  |  |  |  |  |
| ≤48h symptom onset | **0.26 (0.15-0.47)** | **0.24 (0.13-0.43)** | **0.23 (0.09-0.61)** | **0.24 (0.07-0.79)** | **0.17 (0.07-0.43)** | **0.15 (0.06-0.40)** | 0.80 (0.23-2.84)^a^ |  |
| >48h symptom onset | **0.48 (0.30-0.78)** | **0.50 (0.30-0.82)** | 0.63 (0.28-1.39)^b^ | 0.77 (0.28-2.15) | **0.25 (0.12-0.54)** | **0.25 (0.11-0.55)** | 0.83 (0.30-2.32)^c^ |  |
| No | Ref. | Ref. | Ref. | Ref. | Ref. | Ref. | Ref. |  |
| **Seasonal influenza vaccine** | 1.17 (0.83-1.66) |  | 1.02 (0.58-1.79) |  | 1.09 (0.61-1.96) |  | 1.25 (0.54-2.90) |  |

Figures in bold show statistically-significant differences (p<0.05)

Statistical power: ^a^ 3%, ^b^ 18%, ^c^ 6%

COPD: chronic obstructive pulmonary disease
